# Determinants of non-exclusive breastfeeding practice during the first 6 months after an elective caesarean birth: a prospective cohort study

**DOI:** 10.1186/s13006-022-00475-8

**Published:** 2022-05-11

**Authors:** Norkhafizah Saddki, Noraini Mohamad, Nazirah Johar, Tengku Alina Tengku Ismail, Zaharah Sulaiman

**Affiliations:** 1 School of Dental Sciences, Universiti Sains Malaysia, Health Campus, Kelantan, Malaysia; 2 School of Health Sciences, Universiti Sains Malaysia, Kelantan, Malaysia; 3 Department of Community Medicine, School of Medical Sciences, Universiti Sains Malaysia, Health Campus, Kelantan, Malaysia; 4 Women’s Health Development Unit, School of Medical Sciences, Universiti Sains Malaysia, Health Campus, Kelantan, Malaysia

**Keywords:** Non-exclusive breastfeeding, Caesarean birth, Breast milk

## Abstract

**Background:**

Caesarean birth is associated with higher rate of non-exclusive breastfeeding (non-EBF) than vaginal birth. Non-EBF refers to providing food or fluid besides breast milk, excluding drugs and vitamins, to infants before six months of age. This study determined the prevalence and factors associated with non-EBF during the first six months after an elective Caesarean birth.

**Methods:**

This prospective cohort study recruited 171 mothers who underwent an elective caesarean birth at two tertiary hospitals in Kelantan, Malaysia. Face-to-face interviews were conducted two days after the birth to obtain information on the variables of interest. Follow-up phone calls were made at one, three and six months after birth to determine the prevalence of non-EBF. Simple and multiple logistic regressions were used for data analysis.

**Results:**

The prevalence of non-EBF was 19.9%, 40.4% and 57.9% at one, three and six months, respectively. Women who perceived that they had no breast milk, or their breast milk was inadequate were more likely to practise non-EBF at one month [Adjusted Odds Ratio (AOR) 4.83; 95% CI 1.06, 21.96], three months (AOR 4.97; 95% CI 1.67, 14.85) and six months (very often / often AOR 10.06; 95% CI 2.41, 41.99; sometimes / seldom AOR 3.27; 95% CI 1.46, 7.32). Women with at least one child were less likely to practise non-EBF at one month (age of last child ≤ 2 years old AOR 0.10; 95% CI 0.02, 0.66; 3–5 years old AOR 0.10; 95% CI 0.02, 0.53; and > 5 years AOR 0.15; 95% CI 0.02, 0.92).

**Conclusion:**

Perceived breast milk insufficiency was the only factor associated with non-EBF at all time points. The issue of perceived breast milk insufficiency therefore needs to be explored further and addressed by lactation consultants and other maternal and child health professionals. Strategies may include breastfeeding education prior to the surgery and provision of a helpline to provide information and emotional support to the mothers following delivery. The important roles of lactation support groups in early detection and intervention of the problem cannot be emphasised enough.

## Background

Breast milk is the best nutritional source for babies. It is unique and cannot be replaced by other foods, including infant formula or animal milk. Breast milk delivers adequate calories and the necessary proteins, fat, lactose, vitamins, iron, minerals and enzymes in the amounts required for growing infants [1]. Therefore, it is recommended that breastfeeding should be initiated within the first hour after birth and the babies should be breastfed exclusively for the first six months of life [2]. Exclusive breastfeeding (EBF) refers to the act of feeding infants with only breast milk, either directly from their mothers or wet nurses or expressed breast milk, with no added liquids or solids except for syrups consisting of vitamins, mineral supplements or medications [3].

The global rate of EBF was reported as 43% in 2015, a 5% increase over the 38% reported in 2007 [4, 5]. The World Health Assembly Resolution 65.6 endorsed a comprehensive implementation plan on maternal, infant, and young child nutrition that aimed to achieve the rate of EBF in the first six months of at least 50% by 2025. However, if the current rate of EBF is maintained, we will fall short of the 50% global target for the EBF rate by 2025 [6].

The postpartum period can be challenging for nursing mothers. It is even more stressful for mothers who have had a caesarean delivery [7, 8]. Studies have found that women who have given birth via a caesarean section were less likely to breastfeed their babies than those who had a vaginal delivery [9, 10]. Caesarean delivery has also been associated with delayed breastfeeding initiation, shorter breastfeeding duration and a lower rate of EBF than vaginal delivery [11, 12]. A study among Italian women found that elective and emergency caesarean deliveries are similarly associated with a decreased rate of exclusive breastfeeding compared to vaginal birth. They found that only 3.5% of women post-caesarean birth practised breastfeeding compared to 71.5% of women with vaginal birth who breastfed their infants in the birth room [10].

The delayed breastfeeding initiation after a caesarean delivery may be caused by the same factor responsible for delaying skin-to-skin contact between mother and baby after birth, which is the mother’s physical condition after the caesarean delivery [13]. Skin-to-skin contact is defined as the placement of the naked baby on the mother’s bare abdomen or chest immediately or soon after birth for at least one hour or until the baby is ready to breastfeed [14].

Women who have just had a caesarean delivery generally have pain at the surgical site that limitats mobility and causes them difficulty in positioning the baby during breastfeeding. As a result, they may require frequent assistance and a refusal to breastfeed ensues from their frustration [7, 8]. However, with proper support from nurses and doctors, these women will be more likely to breastfeed [15]. It has been shown that breastfeeding education and interventions can effectively increase breastfeeding duration and exclusivity among women [16]. The Ministry of Health of Malaysia recommends strengthening breastfeeding education and support to address specific breastfeeding problems faced by women who have had a caesarean section and can often lead to breastfeeding cessation [17].

Proper planning is the cornerstone of a successful program. In efforts to increase the EBF rate among women after a caesarean delivery, the factors that contribute to their non-exclusive breastfeeding must first be identified. A good understanding of the factors that impede EBF among women after a caesarean delivery will facilitate the development of effective strategic plans that provide breastfeeding education and support for these women to initiate and continue EBF for up to six months. This study aimed to determine the prevalence and factors associated with non-EBF at one month, three months and six months after an elective caesarean delivery in Kelantan, Malaysia. Identification of factors associated with non-EBF at one and three months after caesarean delivery is deemed important because it allows formulation of strategic planning for early intervention to tackle the factors accordingly, hence mothers would be able to sustain EBF for six months, as recommended.

## Method

### Study design and participants

A prospective cohort study was conducted from 1 January 2017 to 31 December 2017 among women admitted for an elective caesarean delivery at two tertiary hospitals in Kelantan, Malaysia. Face-to-face interviews were conducted two days after delivery to obtain information on the variables of interest. Follow-up phone calls were made at one, three and six months after birth to determine the prevalence of non-EBF. Only women admitted for an elective or scheduled caesarean delivery were included in this study. The exclusion criteria were women who had babies born with a congenital anomaly and women who could not initiate breastfeeding due to maternal or neonatal complications. Women diagnosed with psychiatric disorders were also excluded, and this was determined from the medical record.

### Sample size determination and sampling

A convenience sampling method was applied in this study due to the limited sample of mothers who had planned for an elective caesarean delivery. All mothers admitted for an elective caesarean delivery as indicated by an obstetrician during our study period were invited to participate. Among these, 107 (54.9%) participants were recruited from Hospital Universiti Sains Malaysia and 88 (45.1%) from Hospital Raja Perempuan Zainab II. The required number of participants was calculated using the Power and Sample Sizes (PS) Calculation software.The expected proportion of women who had initiated breastfeeding within one hour after delivery among those who practised EBF was estimated at 46.1%, as reported by Tengku Alina et al. [18]. If the proportion of women who had initiated breastfeeding early but did not practise EBF was 68.6%, then we would need a total of 150 participants (75 women who practise non-EBF and 75 women who practise EBF) to be able to reject the null hypothesis with a probability (power) of 0.8. The type I error probability was set at 0.05. In anticipation of a 30% loss in follow-up, a sample size of 195 was decided for this study.

### Research tools

A structured questionnaire was used in this study. The questionnaire was divided into two sections. The first section consisted of three parts. The first part included questions regarding the respondents' demographic characteristics, namely age, race, education level, employment status, and monthly household income. The second part obtained information on previous obstetric and breastfeeding history, current obstetric profile, and time of breastfeeding initiation after caesarean delivery. The third part assessed maternal experience during breastfeeding initiation (the difficulty level in attaching the infant to the breast and placing the infant in the proper breastfeeding position) [7], and maternal experience during breastfeeding practice within 24 h of caesarean section [7, 8, 19].

The first section of the questionnaire was developed based on a review of the literature. Content validity was verified by an expert panel of two lactation consultants and an expert in research methodology. The reliability of the questionnaire was examined by assessing its internal consistency. The questionnaire was found to have good internal consistency based on Cronbach’s α of 0.91 for maternal experience during breastfeeding initiation and 0.71 for maternal experience during breastfeeding practice within 24 h of caesarean section.

The second section of the questionnaire was a feeding practice checklist developed in the Malay language by Muda et al. [20]. The validated checklist contains four questions to ask the mothers if they feed their babies with the following: 1) breastmilk, 2) plain water, 3) formula milk, and 4) complementary food. Three response options, “never”, “seldom”, and “always”, were given to assess the frequency of feeding. The option “seldom” means feeding fewer than seven days per week, and the option "always" means feeding once daily or more. In addition, there is an open-ended question that asks about the main reason for not practising EBF.

### Data collection

Written informed consent was obtained from the women who agreed to participate in the study, and their telephone numbers were also recorded. The study was conducted in two phases. Phase 1 was carried out two days after the elective caesarean delivery when the women were still in the hospital. The first section of the questionnaire was used in this phase of the study. The questionnaire was administered in a face-to-face interview by the main author and a trained research assistant. Information on the patient’s current obstetric profile and caesarean delivery was obtained from the medical records. Phase 2 was conducted at one, three and six months after the caesarean delivery via follow-up phone calls to obtain information regarding the infants’ feeding practices using the second section of the questionnaire.

### Statistical analysis

The analysis started with a data exploration to check the normality of the numerical data and for potential errors in the data entry. The data were presented as mean and standard deviation (SD) for the numerical variables and frequency with percentage (%) for the categorical variables. The data were analysed using simple and multiple logistic regression analyses to determine the factors associated with non-EBF at one month, three months and six months after an elective caesarean delivery.

A simple logistic regression analysis was used to select important variables to be included in multiple logistic regression analysis. Variables with a *P*-value less than 0.25 and variables that were deemed clinically important were included in the subsequent multivariable analysis. This cut point was set at a value larger than the level of significance to obtain as many important variables as possible for selection for inclusion in the model. In the multiple logistic regression analysis, the variables were selected using the forward selection likelihood ratio (LR) method. Following the variable selection, the importance of each variable was verified. The interaction terms were checked using the likelihood ratio test. Multicollinearity problems were identified by the variance inflation factor test. The final model was assessed for fitness using a Hosmer–Lemeshow goodness-of-fit test. The classification table for sensitivity and specificity as well as the area under the receiver operating characteristic (ROC) curve were also obtained. Influential outliers were identified using the Cook’s influence statistics that measure the influence of cases on the predicted values. Data points above 1.0 were considered influential outliers. All statistical analyses were performed with SPSS for Windows version 24.0 (IBM Corp., Armonk, NY, USA). A *P*-value less than 0.05 was considered as statistically significant.

## Results

### Demographic characteristics

A total of 195 women participated and completed the first phase of the study. None of the women were excluded. In Phase 2, only 171 women could be contacted via all of the follow-up phone calls at one, three and six months postpartum to complete the study, providing an 87.6% response rate.

Most of the respondents were Malay (98.8%) and the remaining were Chinese (1.2%). The mean (SD) age of the respondents was 32.3 years (SD 4.83). Nearly half of the respondents had received education up to the secondary (48.5%) and tertiary levels (48.5%), and more than half were employed (57.9%). Most respondents had at least one previous child (79.0%), and the age of the last child for most respondents was five years and below (82.2%). The characteristics of the respondents are shown in Table 1.

**Table 1:** Characteristics of the respondents (*n* = 171)

Variable	Frequency (%)
Age group (years)	
20–30	62 (36.3)
31–40	101 (59.0)
> 40	8 (4.7)
Race	
Malay	169 (98.8)
Chinese	2 (1.2)
Highest education level	
Non formal/Primary	5 (3.0)
Secondary	83 (48.5)
Tertiary	83 (48.5)
Employment status	
Employed	99 (57.9)
Unemployed	72 (42.1)
Parity	
Nulliparous	36 (21.0)
Primiparous/multiparous	135 (79.0)
Age of last child (year) (*n* = 135)	
≤ 5	111 (82.2)
> 5	24 (17.8)

### Previous breastfeeding experience and breastfeeding experience initiation

More than two-thirds of the respondents (78.9%) had breastfed their last child, and almost two-thirds (65.9%) had practised EBF for six months for their last child. Most respondents established skin-to-skin contact with their infants immediately after the caesarean birth (77.8%) and initiated breastfeeding within one hour after birth (73.7%). During the first 24 h following the caesarean birth, almost half of the women (43.8%) perceived their breast milk supply as insufficient. Some also experienced breast symptoms, such as breast pain (43.8%) or a cracked nipple (22.8%).

### Prevalence of non-EBF at one month, three months and six months

The prevalence of non-EBF among the respondents at one month, three months and six months was 19.9%, 40.4% and 57.9%, respectively. The main reason given by most respondents for not practising EBF at each follow-up point was insufficient breast milk.

### Factors associated with non-EBF at one month, three months and six months

The simple logistic regression analysis to determine the factors associated with non-EBF at one month identified 12 variables with *P*-values of less than 0.25. These variables were included in the multiple logistic regression analysis, and three of them were found to be significantly associated with non-EBF at one month. The variables were the age of the last child, breastfeeding practice for the last child and perceived breast milk adequacy (Table 2). These results can be interpreted as follows: 1) women who already had at least one child, regardless of the age of the last child, had lower odds of practising non-EBF at one month than women with no previous child; 2) women who did not practise EBF for their last child had 3.78 times higher odds of non-EBF at one month than women who had practised EBF for six months for their last child and 3) women who had never felt confident that their breast milk was adequate had 4.83 times higher odds of non-EBF at one month than women who very often or often felt confident that their breast milk was adequate.

**Table 2:** Factors associated with NEBF practice at 1 month after an elective caesarean delivery (*n* = 171)

Variable	Crude OR^a^ (95% CI)	Adjusted OR^b^ (95% CI)	Wald^b^ Statistic (df)	*P*-value^b^
Age of last child (year)				
No previous child	1	1		
≤2	0.61 (0.22–1.26)	0.10 (0.02–0.66)	5.76 (1)	0.016
3–5	0.51 (0.21–1.24)	0.10 (0.02–0.53)	7.36 (1)	0.007
> 5	0.66 (0.19–2.25)	0.15 (0.02–0.92)	4.18 (1)	0.041
Last child breastfeeding practice				
EBF for 6 months	1	1		
Did not practice EBF	0.57 (0.19–1.69)	3.78 (1.39–10.29)	6.77 (1)	0.009
No breastfeeding experience	0.86 (0.12–0.67)	0.23 (0.04–1.31)	2.74 (1)	0.098
Felt confident that breast milk is adequate				
Very often/Often	1	1		
Sometimes/Seldom	2.39 (0.98–5.78)	1.63 (0.63–4.22)	0.99 (1)	0.318
Never	7.17 (1.76–29.14)	4.83 (1.06–21.96)	4.15 (1)	0.042

^a^Simple Logistic regression

^b^Multiple logistic regression

*OR=* Odds Ratio

*CI=* Confidence interval

*df=* Degree of freedom

The Hosmer–Lemeshow goodness of fit test *p*-value = 0.676

The percentage of correct classification = 80.1%

The area under ROC curve = 0.739

There are no interaction and multicollinearity problems

The results of the simple logistic regression analysis of the factors associated with non-EBF at three months after an elective caesarean birth identified 17 variables with *P*-values below 0.25, and these were included in the multiple logistic regression analysis. At the multivariate level, the breastfeeding practice for the last child and the perception of having no breastmilk were significantly associated with non-EBF at three months after a caesarean birth (Table 3). The logistic regression model revealed the following: 1) women who did not practise EBF for their last child had 3.72 times increased odds of non-EBF at three months than women who practised EBF for six months for their last child; 2) women who very often or often had a perception of having no breastmilk had 4.97 times increased odds of non-EBF at three months than women who never perceived having no breastmilk.

**Table 3:** Factors associated with NEBF practice at 3 months after an elective caesarean delivery (*n* = 171)

Variable	Crude OR^a^ (95% CI)	Adjusted OR^b^ (95% CI)	Wald^b^ Statistic (df)	*P-*value^b^
Last child breastfeeding practice				
EBF for 6 months	1	1		
Did not practice EBF	0.23 (0.11–0.49)	3.72 (1.69–8.16)	10.69 (1)	0.001
No breastfeeding experience	0.48 (0.18–1.25)	1.81 (0.70–4.67)	1.52 (1)	0.217
Perceived no milk				
Never	1	1		
Sometimes/Seldom	2.27 (1.12–4.61)	1.72 (0.80–3.67)	1.94 (1)	0.164
Very often/Often	7.67 (2.75–21.38)	4.97(1.67–14.85)	8.26 (1)	0.004

^a^Simple Logistic regression

^b^Multiple logistic regression

*OR* =Odds Ratio

*CI=* Confidence interval

*df* =Degree of freedom

The Hosmer–Lemeshow goodness of fit test *p*-value = 0.897

The percentage of correct classification = 69.6%

The area under ROC curve = 0.720

There are no interaction and multicollinearity problems

The simple logistic regression analysis used to predict the factors associated with non-EBF practice at six months after an elective caesarean birth identified 13 variables with *P* -values less than 0.25, and these were included in the multiple logistic regression analysis. At the multivariate level, the factors contributing to non-EBF at six months were: perception of no breastmilk and the experience of breast pain as the baby suckled (Table 4). The logistic regression model revealed the following: 1) women who had a perception that they had no breastmilk had greater odds of non-EBF at six months than women who never had such a perception and 2) women who at any point had an experience of breast pain when the baby suckled had higher odds of non-EBF at six months than women who never had such an experience.

**Table 4:** Factors associated with NEBF practice at 6 months after an elective caesarean delivery (*n* = 171)

Variable	Crude OR^a^ (95% CI)	Adjusted OR^b^ (95% CI)	Wald^b^ Statistic (df)	*P-*value^b^
Perceived no milk				
Never	1	1		
Sometimes/Seldom	2.58 (1.26–5.28)	3.27 (1.46–7.32)	8.35 (1)	0.004
Very often/Often	5.91 (1.88–18.59)	10.06 (2.41–41.99)	10.02 (1)	0.002
Experience breast pain as baby suckled				
Never	1	1		
Sometimes/Seldom	2.35 (1.18–4.68)	2.74 (1.23–6.15)	6.03 (1)	0.014
Very often/Often	3.80 (1.17–12.39)	4.74 (1.09–20.56)	4.31 (1)	0.038

^a^Simple Logistic regression

^b^Multiple logistic regression

*OR=* Odds Ratio

*CI=* Confidence interval

*df* =degree of freedom

The Hosmer–Lemeshow goodness of fit test *p*-value = 0.146

Classification table = 68.4%

The area under ROC curve = 0.724

There are no interaction and multicollinearity problems

## Discussion

In this study, the prevalence of non-EBF among women after an elective caesarean delivery increased as the child's age increased: 19.9%, 40.4% and 57.9% at one month, three months and six months, respectively. The prevalence of non-EBF at one month and three months among women in this study is lower than that reported in the latest National Health Morbidity Survey NHMS, which showed 47.1% and 52.6% at 0 – 2 and 0 – 4 months, respectively, while the prevalence of non-EBF at six months in this study was comparable to the national rate of 52.9% among infants below six months old [17]. In Italy, a study among 398 women who delivered by elective caesarean also showed comparable findings, indicating that 25.5%, 44.9% and 53.4% of the women discontinued EBF at seven days, three months and six months, respectively [10]. A six -month cohort study in Hunan, China reported higher non-EBF rates for woman after a caesarean delivery, with 28.7% and 79.8% of the mothers discontinuing EBF at one month and six months, respectively. However, at three months after a caesarean delivery, a finding similar to the current study was reported, with 40% of the mothers in Hunan discontinuing EBF [11].

In our study, women who already had at least one child, regardless of the age of the last child, were more likely to continue EBF for one month after a caesarean delivery than those who did not have any children. This finding may be due to first-time mothers having less awareness of the advantages of breastfeeding as compared to multigravida women [21]. It has been reported that early breastfeeding problems and mixed feeding practices at the time of hospital discharge are more common among primiparous women than multiparous women. Multiparous mothers have also shown significantly longer breastfeeding duration than primiparous mothers [22].

A positive attitude to EBF is more prevalent among multiparous mothers, most probably due to their higher maternal breastfeeding self-efficacy, in which the mother feels confident in her ability to breastfeed. The self-efficacy also increases with the past successful experience and performance [23]. Besides previous positive breastfeeding experience, women can also develop their self-efficacy through the exposure to vicarious experiences (such as observing other mothers who breastfeed), watching videos related to breastfeeding, receiving verbal persuasion and encouragement from their friends and family, and the generation of pleasant and positive feelings towards breastfeeding [24]. Therefore, strategies to increase the EBF rate among primiparous women should focus on improving self-efficacy for continuing EBF and, subsequently, help them deal with any breastfeeding challenge [22].

The women who did not practise EBF for their last child were more likely to discontinue EBF at one month and three months after a caesarean delivery. Our findings are in agreement with a study in Hong Kong which showed that the participants who did not breastfeed exclusively or who practised EBF for up to only two months were more likely to stop EBF earlier than those who breastfed exclusively for more than two months [25]. A systematic review on breastfeeding experiences concluded that previous breastfeeding experience consistently correlates with subsequent breastfeeding initiation and duration. Previous short breastfeeding duration and unsatisfactory experience can negatively affect subsequent breastfeeding practices [26]. A qualitative study in the United States found that women who had successfully breastfed in the past were intrinsically motivated from their own emotional attachment to the practice. Besides, they were also extrinsically motivated from their family who encouraged them to breastfeed [27]. Therefore, it is recommended for midwives and lactation consultants to provide individualised interventions to the mothers based on their previous breastfeeding experience to improve breastfeeding initiation and duration [26].

In our study, the women who had never felt confident that their breast milk was adequate or who had at any point perceived that they had no breast milk were more likely to discontinue EBF within the first six months after a caesarean delivery. A perception of insufficient milk is defined as the mother's perception that she is not producing an adequate supply of breast milk to meet her infant's needs [28]. Other studies in Vietnam, Taiwan and Australia have noted the same perception which is the most common reason the mothers discontinued EBF [29–31]. It could be related to the low level of maternal self-confidence in the ability to breastfeed. This fact is further supported by a study by Blyth et al. which revealed that the mothers with a high breastfeeding self-efficacy were significantly more likely to practise EBF at one week and four months postpartum than the mothers with low breastfeeding self-efficacy [31]. Support and reassurance from hospital staff are very crucial at this stage. Mothers should also be educated on how to assess their breastmilk adequacy so that they will be able to distinguish between a perceived breastmilk insufficiency and a true breastmilk insufficiency. The best way to assess breastmilk supply is by monitoring infant weight gain and measuring stool output, and this can be managed with patient education, support and reassurance [32].

Breastfeeding difficulties due to breast conditions such as sore or cracked nipples or breast engorgement commonly occur during the first few days of breastfeeding**.** These conditions can arise due to improper latching or improper positioning of the baby during feeding [32]. In our study, mothers who experienced breast pain as the baby suckled were more likely to discontinue EBF at six months after a caesarean delivery. A study in Kelantan, Malaysia showed that sore or cracked nipples, difficulty with latching and breast engorgement were associated with the discontinuation of EBF at one month [18]. On the other hand, a study done among women in Argentina found that mothers who had no nipple problems and whose child had an appropriate suckling technique were more likely to practise EBF for a longer duration [33]. Breastfeeding difficulties that persist beyond the first few days after birth can be discouraging and may lead to early discontinuation of EBF. With the right help, however, most of these difficulties can be overcome.

In this study, none of the mothers’ socio-demographic characteristics, including age, educational level or employment status, were significantly associated with their non-EBF during the first six months after an elective caesarean delivery. On the contrary, a multilevel analysis on the factors associated with non-EBF in five Asian countries found that first-born infants, working mothers and higher maternal age were the significant individual factors associated with non-EBF [34]. Another study in Ethiopia reported that mothers who completed primary school were less likely to practise non-EBF as compared to mothers with no formal education, while governmental employees were more likely to practise non-EBF [35].

The strength of the current study was that we identified factors that contributed to the practice of non-EBF at one, three and six months after an elective caesarean delivery. This important information provides a basis on which strategic planning can be made. Early intervention such as exposure to breastfeeding educational modules to support post-caesarean women in the postnatal wards may help them overcome the factors contributing to non-EBF at one month and three months, such that the women would be able to sustain EBF for six months, as recommended.

In addition, the educational interventions should also be delivered to women during the antenatal period so that they are well-informed about the circumstances they may encounter during the initiation and maintenance of EBF, including perceived breast milk insufficiency and breast pain. As a result, they will be physically and mentally well-prepared to face the challenges. The interventions should include information on different types of breastfeeding positions that are convenient for the women after a caesarean delivery. They should also provide information on how to increase breast milk production since a perceived breast milk insufficiency is the only factor associated with non-EBF practice at all months.

Furthermore, mothers must be informed about where to seek help if they do face problems related to breastfeeding after being discharged from the hospital. This can be done by providing them the lactation helpline number, by which the necessary breastfeeding advice and guidance can be offered. Mothers should also be encouraged to join the lactation support group to discuss their breastfeeding problems with other women who have had an elective caesarean and share their solutions to those problems.

This study has several limitations. The findings of this study cannot be inferred to all women admitted for an elective caesarean delivery in Malaysia as the sample was restricted to only two tertiary hospitals in Kelantan, Malaysia and women were recruited using convenience sampling. Furthermore, this study was carried out at hospitals located in an area where Malays are the predominant ethnic group. The results are thus not generalisable to other racial groups or settings. Therefore, replicating this study in a larger, more racially or ethnically diverse sample should be considered.

## Conclusion

In this study, perceived breast milk insufficiency, lack of previous experience with EBF, having no previous child and the experience of breast pain while breastfeeding were all associated with non-EBF among women during the first six months after an elective caesarean delivery. Perceived breast milk insufficiency was the only factor associated with non-EBF at all months. Thus, mothers must be taught how to assess milk adequacy and maintain or increase breast milk production. Mothers also need to be convinced that their breast milk is adequate and that they should continue breastfeeding. Health professionals and lactation consultants play the important roles in encouraging mothers to initiate breastfeeding and maintain EBF for the first six months after a caesarean delivery.

## Data Availability

Data used in this study are available upon reasonable request. Please contact the corresponding author for data requests.

## Abbreviations

AOR: Adjusted odds ratio
CI: Confidence interval
EBF: Exclusive breastfeeding
non-EBF: Non-exclusive breastfeeding
